# Synchronous inhibition of mTOR and VEGF/NRP1 axis impedes tumor growth and metastasis in renal cancer

**DOI:** 10.1038/s41698-019-0105-2

**Published:** 2019-12-05

**Authors:** Krishnendu Pal, Vijay Sagar Madamsetty, Shamit Kumar Dutta, Enfeng Wang, Ramcharan Singh Angom, Debabrata Mukhopadhyay

**Affiliations:** Department of Biochemistry and Molecular Biology, Mayo Clinic Florida, 4500 San Pablo Road S, Jacksonville, FL 32224 USA

**Keywords:** Targeted therapies, Targeted therapies, Renal cell carcinoma

## Abstract

Clear cell renal cell carcinoma (ccRCC) is known for its highly vascular phenotype which is associated with elevated expression of vascular endothelial growth factor A (VEGF), also known as vascular permeability factor (VPF). Accordingly, VEGF has been an attractive target for antiangiogenic therapies in ccRCC. Two major strategies have hitherto been utilized for VEGF-targeted antiangiogenic therapies: targeting VEGF by antibodies, ligand traps or aptamers, and targeting the VEGF receptor signaling via antibodies or small-molecule tyrosine-kinase inhibitors (TKIs). In the present article we utilized two entirely different approaches: targeting mammalian target of rapamycin (mTOR) pathway that is known to be involved in VEGF synthesis, and disruption of VEGF/Neuroplin-1 (NRP1) axis that is known to activate proangiogenic and pro-tumorigenic signaling in endothelial and tumor cells, respectively. Everolimus (E) and a small-molecule inhibitor EG00229 (G) were used for the inhibition of mTOR and the disruption of VEGF/NRP1 axis, respectively. We also exploited a liposomal formulation decorated with a proprietary tumor-targeting-peptide (TTP) to simultaneously deliver these two agents in a tumor-targeted manner. The TTP-liposomes encapsulating both Everolimus and EG00229 (EG-L) demonstrated higher in vitro and in vivo growth retardation than the single drug-loaded liposomes (E-L and G-L) in two different ccRCC models and led to a noticeable reduction in lung metastasis in vivo. In addition, EG-L displayed remarkable inhibition of tumor growth in a highly aggressive syngeneic immune-competent mouse model of ccRCC developed in Balb/c mice. Taken together, this study demonstrates an effective approach to achieve improved therapeutic outcome in ccRCC.

## Introduction

Clear cell renal cell carcinoma (ccRCC) is the most prevailing (75–80%) subtype of RCC, which, in turn, accounts for approximately 90% of all kidney cancers.^1,2^ The von Hippel-Lindau (VHL) tumor suppressor gene is often inactivated in ccRCC, leading to the stabilization and consequent accumulation of hypoxia-inducible-factor-1α (HIF-1 α)^3^ and overexpression of VEGF (also known as VPF).^4,5^ This VHL gene inactivation and high VEGF expression in RCC have been correlated with tumor aggressiveness and poor survival.^6^ VEGF plays a critical role in both normal and tumor-associated angiogenesis via stimulation of endothelial cell proliferation and migration,^7^ protection of endothelial cells from apoptosis,^8^ and reversal of senescence in endothelial cells.^9^ VEGF is known to exert its effect through the interaction with transmembrane tyrosine-kinase receptors that include VEGF receptor 1 (VEGFR1), VEGF receptor 2 (VEGFR2), and Neuropilin-1 (NRP1).^10^ Naturally, all of these receptors are expressed in vascular endothelial cells. However, NRP1 is also expressed in other cells including tumor cells^11^ and immune cells.^12^ Although, VEGFR2 is well established as the key receptor behind the proangiogenic signaling of VEGF,^13^ NRP1 has been shown to play an essential role in VEGF induced endothelial cell migration.^14^ In addition, several recent studies implicated VEGF/NRP1 axis in tumor-cell autocrine signaling pathways responsible for imparting cancer stemness.^15–20^

With the advent of the concept of antiangiogenic therapy,^21^ targeting VEGF became a lucrative option for the treatment of cancer, especially in highly angiogenic types such as ccRCC. Majority of hitherto used VEGF-targeted antiangiogenic therapies fall in two main categories.^22^ The first approach targets VEGF directly utilizing monoclonal antibodies, soluble receptor/ligand traps or aptamers, while the other strategy inhibits VEGF signaling by targeting VEGFR2 with monoclonal antibodies or small-molecule tyrosine-kinase inhibitors (TKIs). Among the clinically approved VEGF-targeting agents, the humanized anti-VEGF monoclonal antibody Bevacizumab falls under the first category while the small-molecule VEGFR-TKIs such as Sorafenib and Sunitinib are examples of the latter. Initially, these treatment regimens provided a paradigm shift for the treatment of ccRCC. However, patients ultimately became resistant towards the antiangiogenic therapies, thus limiting the long-term benefits.^23^

In addition to the above anti-VEGF treatments, several other routes have been evaluated for antiangiogenic therapy. For instance, mTOR inhibitors have been shown to inhibit hypoxia- or growth factor-induced endothelial cell proliferation, migration, and tube formation in in vitro studies.^24^ Likewise, the antiangiogenic efficacy of mTOR inhibitors have been validated in a variety of cancer models in vivo.^25–28^ Inhibition of mTOR induces apoptosis in tumor-associated endothelial cells that ultimately leads to significant reductions in microvessel density and tumor growth. In addition, mTOR inhibitors can also impact angiogenesis by inhibiting the production of proangiogenic factors in tumors and tumor-associated stromal cells.^29^ Moreover, mTOR contributes in the hypoxic tumor response by stabilizing hypoxia-inducible factor-1α (HIF-1 α),^30^ hence it is not surprising that mTOR inhibitors can reduce VEGF expression and act as antiangiogenic agents.^31^

Recently, several articles described the use of VEGF/NRP1 axis inhibitors, ranging from peptide fragments to small-molecule inhibitors, as an alternative therapeutic strategy for cancer.^32–38^ Among them, one small-molecule inhibitor, EG00229, demonstrated remarkable growth inhibition in glioma and lung cancer via combined antiangiogenic and antitumor activity.^36,37^ In addition, EG00229 has been shown to elicit immune-modulatory activity by blocking the M2 shift in microglial cells.^39^ Based on our previous work showing that the VEGF/NRP1 axis can be a great target for ccRCC,^16^ we sought to examine whether EG00229 would have similar antitumor efficacy in RCC, either alone or in combination with other drugs.

Hence, in the present work, we proposed to target the mTOR pathway with Everolimus (E) and simultaneously disrupt the VEGF-NRP1 axis by using EG00229 (G) that might block both VEGF/VEGFR2/NRP1-mediated proangiogenic signaling pathways in endothelial cells and VEGF/NRP1/Ras-mediated autocrine activation of tumor cell growth.^16^ We assumed that by doing so, our strategy will reduce the amount of available VEGF and, at the same time, inactivate the residual VEGF from activating its proangiogenic and pro-tumorigenic downstream signaling pathways. Since both Everolimus and EG00229 are water-insoluble compounds, we developed a liposomal formulation entrapping both for easy and efficient delivery, instead of using complex delivery vehicles consisting of DMSO, ethanol, surfactants, or polyethylene glycol. Another added advantage of our liposomal formulation is that we have tagged a proprietary tumor-targeting peptide (TTP) to the surface of the liposomes to enhance the tumor-specific delivery of the drugs and reduce any toxicity arising from the treatment of Everolimus as well as systemic inhibition of NRP1. Here, we report the antitumorigenic and anti-metastatic efficacy of the dual-drug-loaded liposomal formulation EG-L in two different ccRCC xenograft models and the immune-modulatory effect of EG-L in a highly aggressive immune-competent syngeneic mouse model of ccRCC.

## Results

### Synthesis and characterization of liposomes

The amount of lipid and drug components of empty liposomes (L) and drug-loaded liposomes (E-L, G-L, and EG-L) are reported in Table 1 along with drug-loading efficiency (DLE) and encapsulation efficiency (EE) values where applicable. The initial amounts of both Everolimus and EG00229 used during preparation of liposomes were 0.4 mg per 1 mL of liposomes, respectively. Everolimus, being highly water-insoluble lipophilic drug, displayed an EE of ~100% (concentration in liposome = 0.4 mg/mL) due to its complete incorporation in the liposome bilayer. EG00229, initially being in the aqueous portion, displays only 30% EE (concentration in liposome = 0.12 mg/mL) due to its comparatively higher hydrophilic nature. The DLE of Everolimus in E-L and EG00229 in G-L were 7.29% and 2.2% respectively. The DLE and EE values in dual-drug-loaded liposomes (EG-L) did not show any alterations from the single drug-loaded ones. Plausibly, the distinct spatial distribution of Everolimus and EG00229 inside the liposomes is responsible for this observation. A higher DLE will require fewer amounts of carrier lipids to deliver the same amount of drugs thus increasing the efficacy of the treatments whereas a higher EE would ensure minimum loss of drugs during the preparation of the liposomal formulations. The DLE and EE of EG-L are more or less comparable with the values reported for the hydrophobic and hydrophilic drugs in literature.^40^

**Table 1:** Encapsulation efficiency (EE) and drug loading efficiency (DLE) of the liposomes.

Liposome	DOPC (mg/mL)	Cholesterol (mg/mL)	DSPE(PEG)2000-OMe (mg/mL)	TTP (mg/mL)	E (mg/mL)	G (mg/mL)	DLE (%)	EE (%)
L	3.93	0.965	0.140	0.452	–	–	–	–
E-L	3.93	0.965	0.140	0.452	0.4	–	7.29	100
G-L	3.93	0.965	0.140	0.452	–	0.120	2.2	30
EG-L	3.93	0.965	0.140	0.452	0.4	0.120	7.29 (E), 2.2 (G)	100 (E), 30 (G)

Encapsulation efficiency and drug loading efficiency of liposomes containing Everolimus (E-L), EG00229 (G-L), and combination of Everolimus and EG00229 (EG-L), *n* = 1 measurement per sample

The average hydrodynamic size, polydispersity index (PDI), and zeta potential of empty liposomes (L) as well as liposomes containing Everolimus (E-L), EG00229 (G-L), and a combination of both (EG-L) are consolidated in Table 2. The entrapment of drugs caused mostly minor changes in the size and PDI of the liposomes except for encapsulation of EG00229 (G-L) where the size and PDI of the liposomes increased significantly. Nonetheless, all the liposomal formulations had average size of less than 100 nm which is suitable for better penetration through tumor microenvironment.^41^ However, the zeta potentials were significantly different among the liposomes. The empty liposomes had a zeta potential of 32.9 ± 2.3 mV (± values are given based on standard deviations, *n* = 3). Encapsulation of Everolimus decreased the zeta potential to 18.3 ± 1.7 mV whereas encapsulation of EG00229 decreased it to 22.4 ± 4.3 mV. The liposomes encapsulating both the drugs had a higher zeta potential (47.53 ± 1.6 mV) than other liposomes. A highly positive zeta potential indicates stability of the liposomal suspension as well as stronger interaction with negatively charged cell membranes.^42^ Since all of our liposomes were positively charged, we expect these formulations to be stable and efficient in cellular uptake.

**Table 2:** Characterization of liposomal drug formulations.

Liposome	Size (nm)	PDI	Zeta (mV)
L	67.1 ± 0.12	0.24 ± 0.05	32.9 ± 2.3
E-L	62.15 ± 0.4	0.18 ± 0.01	18.3 ± 1.7
G-L	95.35 ± 0.69	0.334 ± 0.01	22.4 ± 4.3
EG-L	71.86 ± 0.16	0.351 ± 0.01	47.53 ± 1.6

Hydrodynamic size, polydispersity index (PDI), and zeta potential of liposome only (L), or liposomes containing Everolimus (E-L), EG00229 (G-L), and a combination of Everolimus and EG00229 (EG-L). All the measurements were performed in deionized water at 25 °C. ± values are based on standard deviations, *n* = 3 measurements per sample

### In vivo biodistribution of liposomes in ccRCC xenograft bearing mice

**Fig. 1:**
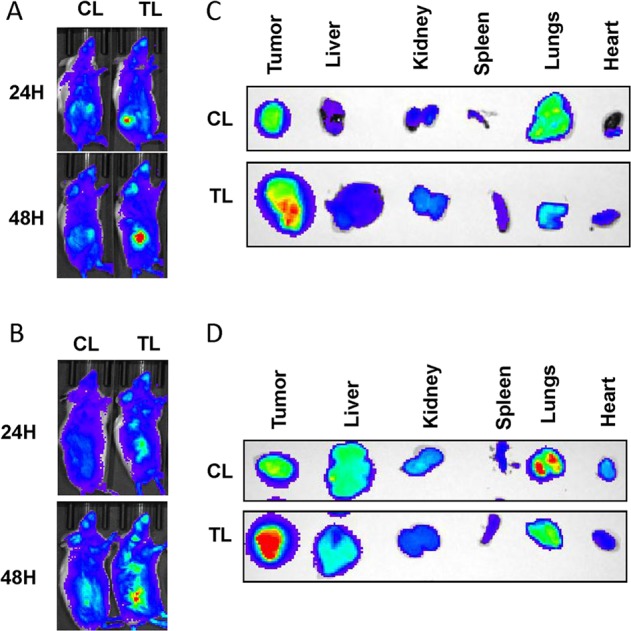
In vivo biodistribution of IR-780-dye-labeled liposomes in RCC xenografts. IVIS imaging showing higher tumor accumulation of IR-780 dye-labeled TTP-conjugated liposomes (TL) compared to control liposomes (CL) at 24 h (upper panel) and 48 h (lower panel) after IV administration into mice bearing subcutaneous 786-O (**a**) and A498 tumors (**b**). Ex vivo imaging of 786-O (**c**) and A498 (**d**) tumors and major organs, respectively, harvested at 48 h, demonstrated significant higher tumor uptake of TL compared to CL. Interestingly, significantly higher lung accumulation of CL was observed compared to TL. *n* = 1 mouse per treatment group.

After performing the physical characterizations, we decided to examine the in vivo tumor-targeting efficacy of these liposomes in ccRCC tumor bearing mice. Hence, we performed biodistribution studies in subcutaneous 786-O or A498 xenografts after intravenous administration of IR-780-dye labelled liposomes. We used both TTP-conjugated liposomes (TL) and control liposomes without any TTP (CL) in these experiments. IR-780-dye was used as its excitation and emission peaks reside in the IR region of the spectrum resulting minimal loss of intensity from absorption by live tissue. In addition, no discernible autofluorescence from mouse fur interfering with the signal intensity was witnessed in this region. As expected, TL resulted in higher tumor-specific signals than CL at 24 and 48 h post administration in both 786-O and A498 xenografts (Fig. 1a, b), which was further supported by the ex vivo imaging of the tumors and major organs (Fig. 1c, d). In addition, lungs from CL-treated mice displayed stronger signal than lungs of TL-treated mice, which indicates that TTP is more effective in diminishing the nonspecific accrual of the liposomes in the lungs.

### In vitro efficacy of drug-loaded liposomes in ccRCC

**Fig. 2:**
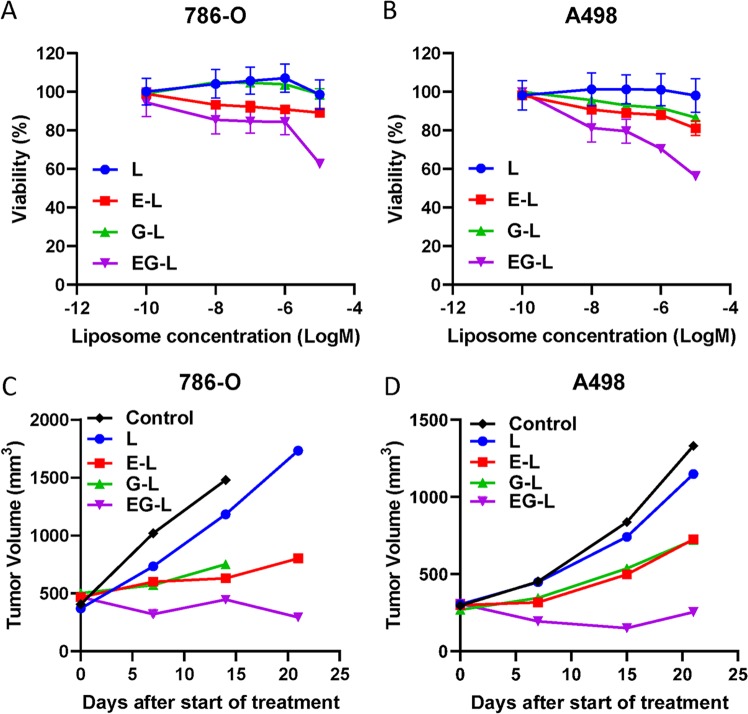
In vitro and in vivo efficacy of drug-loaded liposomes in RCC cell lines. 786-O (**a**) and A498 (**b**) cells were treated with various drug-loaded TTP-conjugated liposomes for 72 h. Then cell viability was determined with MTS assay. Dual-drug-loaded liposomes showed higher reduction in cell viability compared to single drug-loaded liposomes in all cell lines (*n* = 4 wells per dose). **c** 5 × 10^6^ 786-O cells were subcutaneously injected into the right flanks of 8-week-old male SCID mice. Tumors were allowed to grow until the average tumor size is ~400–500 mm^3^. Then mice were treated with drug-loaded liposomes (*n* = 1 mouse per treatment group) 3× per week for 3 weeks. Tumors were measured weekly and tumor volume was plotted to obtain the respective growth curves. In both cases dual-drug-loaded liposomes demonstrated stronger inhibition compared to single drug-loaded liposomes. Some of the mice were sacrificed before the completion of experiment due to ulceration of tumors. **d** Similar results were obtained in A498 xenografts (*n* = 1 mouse per treatment group).

On the basis of the above biodistribution study, we used TL in all further efficacy studies since our goal was to exploit the tumor-targeting ability of TL for precision therapy. However, before going for animal studies, we examined the drug-loaded liposomal formulations for their in vitro efficacy in 786-O and A498 cells. As shown in Fig. 2a, b, EG-L was more effective in reducing cell viability than E-L or G-L in both 786-O and A498 cell lines.

### In vivo efficacy of drug-loaded liposomes in ccRCC xenografts

We then proceeded to analyze the in vivo efficacy of the drug-loaded liposomes in two different ccRCC xenografts in immune-deficient mouse models and one immune-competent syngeneic mouse model. In the initial screening experiments, we employed the single mouse trial (SMT) strategy, a lately popularized concept that has been well accepted by scientific community.^43–45^ A single mouse per treatment arm is employed in this approach to reliably detect the most effective treatment from a large number of treatment regimens in a cost-effective manner by analyzing the longitudinal growth of each tumor. Understandably, SMT is not expected to provide the statistical significance of the observed result; however, this limitation may be alleviated by performing a validation study in larger cohorts with the most effective treatment.

**Fig. 3:**
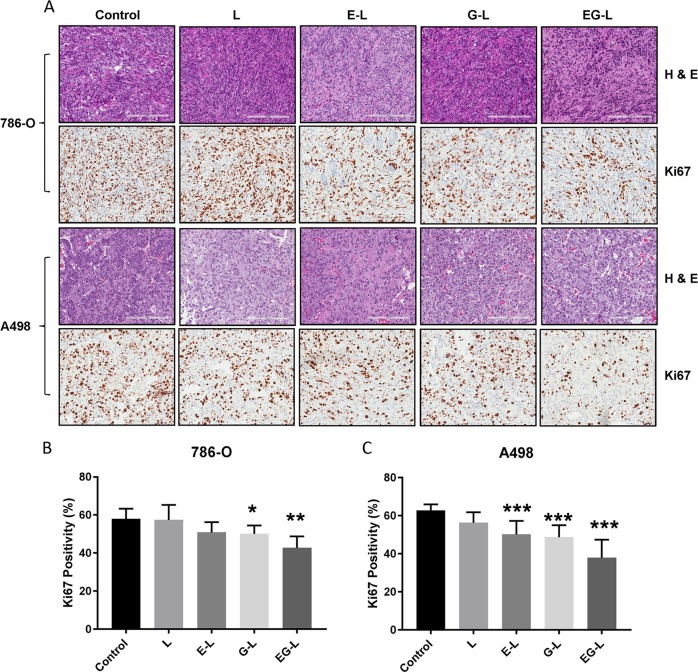
H&E and Ki67 staining of tumor sections obtained from the single mouse trial. **a** Representative images of the H&E and Ki67 stained tumor sections from different treatment groups displayed comparatively higher antiproliferative activity of EG-L. Bar length = 200 µm. **b**, **c** Quantification of Ki67-positive nuclei in 786-O and A498 tumor sections, respectively. Error bars in all graphical plots are given based on standard deviation. *, ** and *** denote *p* < 0.05, *p* < 0.01, and *p* < 0.001 compared to control, respectively (*n* = 5 spatially different regions from same tumor section).

In our experiments, we allowed the tumors to grow larger before starting the treatment to challenge our drug-loaded liposomal formulations against comparatively worse pathological conditions. In most of our experimental design, a starting tumor volume of 300–500 mm^3^ has been used, which is considerably higher than the usual 50–100 mm^3^ volumes commonly reported in tumor growth retardation studies. Similar to the in vitro studies, EG-L was better than E-L or G-L in impeding tumor growth in both 786-O and A498 xenografts (Fig. 2c, d). Importantly, EG-L demonstrated discernible reductions in tumor volumes from the initial higher values in both the tumor models, which suggests its superior antitumorigenic efficacy. This was further substantiated from the H&E and Ki67 staining of the tumor sections (Fig. 3a–c) that exhibited significantly higher antiproliferative activity of EG-L than that of E-L or G-L. In addition, liver, kidney, and spleen did not show any significant changes in gross morphology, suggesting that those organs were not adversely affected by the drug-loaded liposome treatment (Supplementary Figs S1 and 2).

**Fig. 4:**
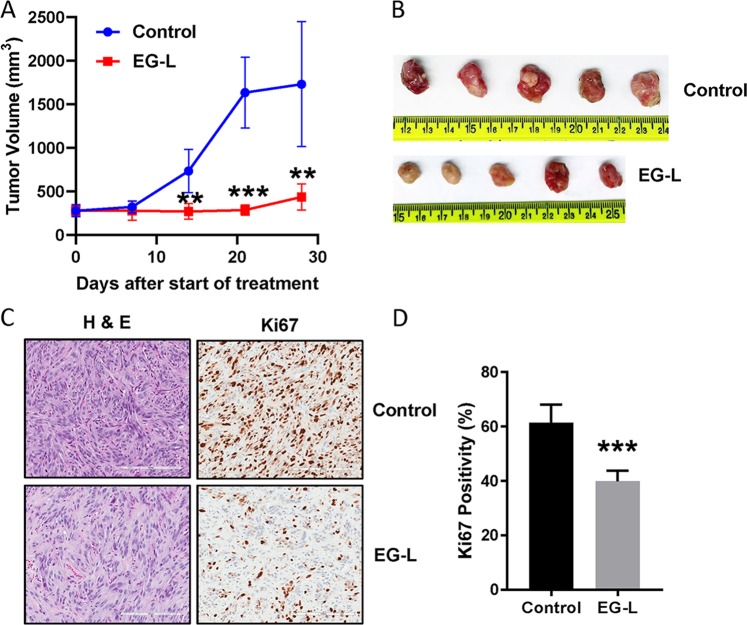
Validation of the result obtained from single mouse trial in cohorts of five mice. **a** 5 × 10^6^ 786-O cells were subcutaneously injected into the right flanks of 8-week-old male SCID mice. Tumors were allowed to grow until the average tumor size is ~300 mm^3^. Then mice were treated with vehicle or EG-L (*n* = 5 mice per treatment group) 3× per week for 4 weeks. Tumors were measured weekly and tumor volume was plotted to obtain the respective growth curves. EG-L demonstrated significant inhibition compared to the vehicle group. ** and *** denote *p* < 0.01 and *p* < 0.001 compared to control, respectively. **b** Images of the harvested tumors at the end of the experiment. **c** Representative images of H&E and Ki67 staining of the tumor tissue sections. Bar length = 200 µm. **d** Quantification of Ki67-positive nuclei. *** denotes *p* < 0.001 compared to control (*n* = 3 tumors per group, five spatially different regions from each tumor section).

We further performed a validation study in cohorts of five mice bearing 786-O xenografts to confirm whether the potent antitumorigenic efficacy of EG-L is truly reproducible and statistically significant. We obtained essentially similar results to the SMT demonstrating noticeable and statistically significant reductions in tumor volumes from higher starting values up to 3 weeks of treatment (Fig. 4a–d). In a way, this result corroborates the relevance of the SMT in recognizing the optimal treatment strategy for combating cancer.

**Fig. 5:**
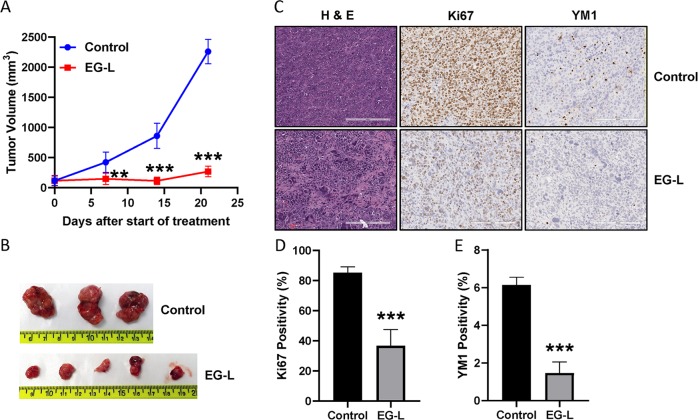
Antitumor efficacy of EG-L in an immune-competent mice model of RCC. **a** 1 × 10^6^ Renca cells were subcutaneously injected into the right flanks of 8 week-old-male Balb/c mice. Tumors were allowed to grow until the average tumor size is ~120 mm^3^. Then mice were treated with vehicle or EG-L (*n* = 5 mice per treatment group) 2× per week for 3 weeks. Tumors were measured weekly and tumor volume was plotted to obtain the respective growth curves. EG-L demonstrated significant inhibition compared to the vehicle group. ** and *** denote *p* < 0.01 and *p* < 0.001 compared to control, respectively. **b** Images of the harvested tumors at the end of the experiment. Two tumors from control groups were ruptured before harvest. **c** Representative images of H&E, Ki67, and YM1 staining of the tumor tissue sections. Bar length = 200 µm. **d**, **e** Quantification of Ki67 and YM1 staining respectively. *** denotes *p* < 0.001 compared to control (*n* = 3 tumors for control and 4 tumors for EG-L, five spatially different regions from each tumor section).

We also analyzed the efficacy of EG-L in a highly aggressive syngeneic mouse ccRCC model developed by subcutaneous implantation of Renca cells in immune-competent Balb/c mice. This ccRCC model is resistant to immune therapy by anti-PD-1 antibody or small-molecule inhibitor of PD-1/PD-L1 interaction (Supplementary Fig. S3A, B) and therefore mimics ~80% of ccRCC patients who do not respond to immune therapy. Although EG-L was not able to reduce the tumor volume from initial value in this highly aggressive model, it displayed remarkable tumor growth retardation over the course of the study (Fig. 5a, b). The H&E and Ki67 staining of the tumor sections demonstrated strong antiproliferative activity (Fig. 5c, d). In addition, a marked reduction in YM1 positivity in EG-L-treated tumor sections was observed (Fig. 5c, e). YM1 is a marker of macrophage M2 polarization, which is usually responsible for suppression of antitumor immunity and increase in tumor growth. Hence, there may be a plausible immune-modulatory role of EG-L behind its strong antitumor response in this model. Interestingly, we did not observe any additive or synergistic effect when EG-L was combined with anti-PD-1 antibody or small-molecule inhibitor of PD-1/PD-L1 interaction (Supplementary Fig. S3A, B). Furthermore, we observed no significant change in the expression of PD-L1 or PD-1 in EG-L-treated tumors compared to control although PD-1 expression was slightly higher in the EG-L-treated group (Supplementary Fig. S4A-D). The uncropped scans of the blots are provided in Supplementary Fig. S5.

**Fig. 6:**
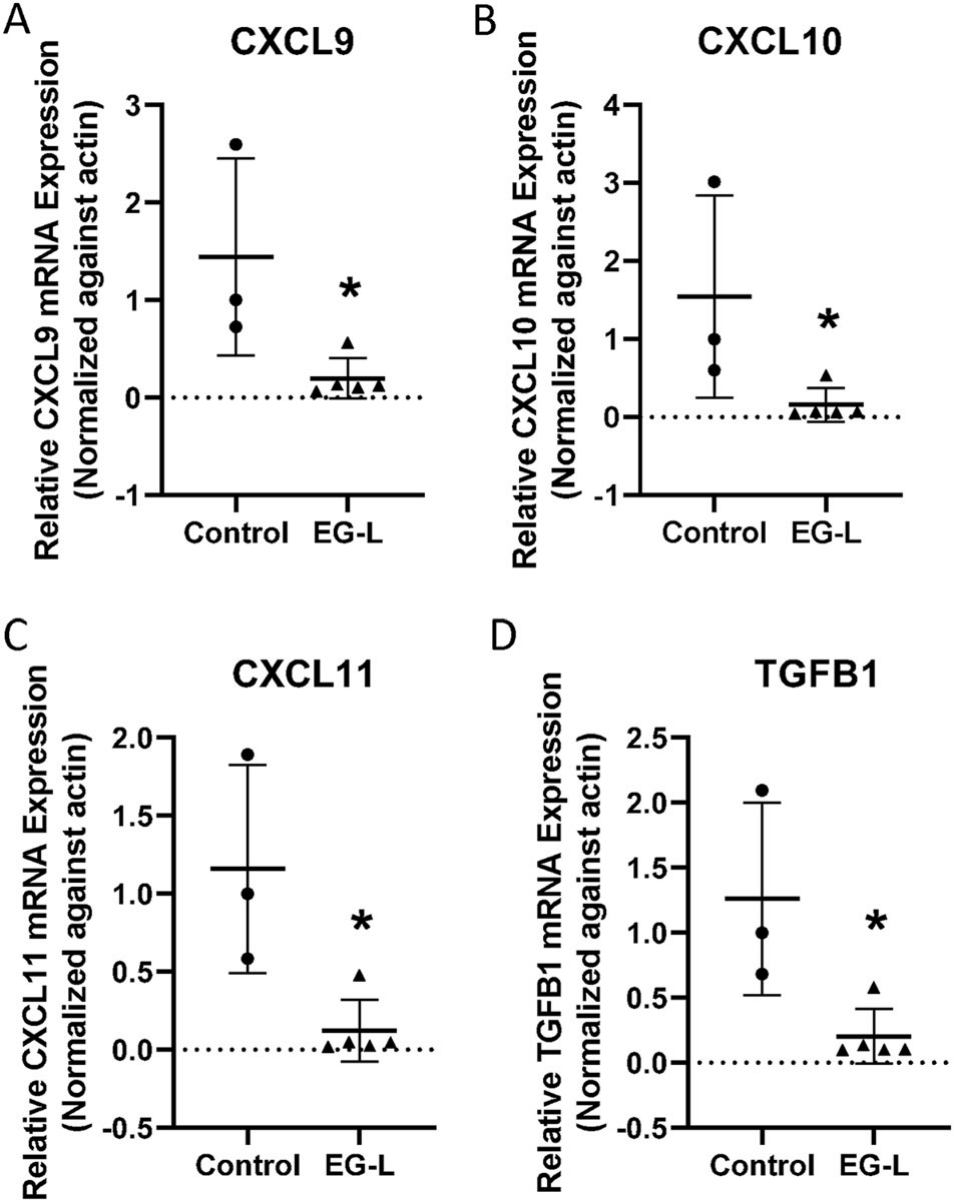
EG-L downregulates the expression of C-X-C chemokines and TGF-β in an immune-competent mice model of RCC. Total RNA was isolated from tumors treated with vehicle or EG-L and subjected to real-time reverse transcription polymerase chain reaction (RT-PCR) for **a** CXCL9, **b** CXCL10, **c** CXCL11, and **d** TGFB1. EG-L significantly reduced the mRNA expression of the cytokines compared to the vehicle. * denotes *p* < 0.5 compared to control. (*n* = 3 tumors for control and 5 tumors for EG-L).

We also tested if EG-L treatment led to any alterations in chemokine and cytokine expression that are known modulators of immune infiltration. As depicted in Fig. 6, we observed significant reductions in mRNA levels of C-X-C motif chemokine receptor 3 (CXCR3)-associated chemokines C-X-C Motif Chemokine Ligand-9 (CXCL9), C-X-C Motif Chemokine Ligand-10 (CXCL10), and C-X-C Motif Chemokine Ligand-11 (CXCL11). TGFB1 mRNA levels were reduced as well, which corroborates with previous studies.^38^

### Inhibition of lung metastases

**Fig. 7:**
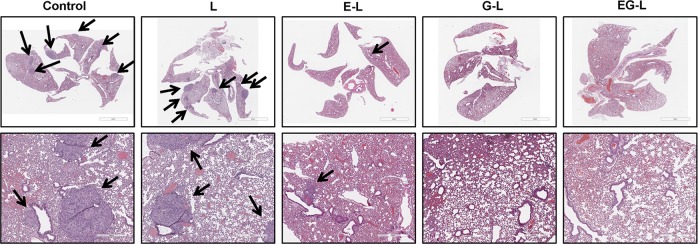
Inhibition of lung metastasis in mice bearing 786-O xenografts. The dual-drug-loaded liposomes (EG-L) significantly inhibited lung metastasis in mice bearing 786-O subcutaneous tumors compared to Control, liposome only (L), or single-drug-loaded liposomes (E-L and G-L). Metastatic nodules are indicated by black arrows (upper panel). Respective higher magnification images are depicted in the lower panel (*n* = 1 mouse per treatment group).

Since ccRCC is notorious for inducing lung metastases,^46^ we further explored the efficacy of our drug-loaded liposomal formulations in reducing the metastatic burden. Interestingly, the H&E-stained whole-lung sections displayed metastatic nodules in the control mouse or mice treated with L or E-L, whereas G-L and EG-L treated mice lung sections exhibited no detectable nodules (Fig. 7). Our results suggest that both G-L and EG-L were capable of reducing the lung metastases in ccRCC, which might have significant clinical relevance.

## Discussion

Kidney cancer is the sixth and eighth most common cancer in men and women in the United States, respectively, and in 2019, an estimated 73,820 new diagnoses and 14,770 deaths are projected in the United States related to this desease.^47^ Approximately 90% of all kidney cancers are diagnosed as RCC.^1^ RCC has three major histologic subtypes: ccRCC (75–80%), papillary RCC (10%-16%), and chromophobe RCC (5%).^2^ While prognosis is usually better in patients with early stage RCC, advanced metastatic RCC is a life-threatening disease having a meagre 11.7% 5-year survival rate. Approximately one-third of RCC patients have already developed metastasis at diagnosis and distant metastases is observed in up to 50% patients after resection of primary tumor.^48^ Conventional chemotherapy and radiation therapy are mostly futile in RCC.^49,50^ Current first- and second-line treatments for RCC consisting of tyrosine-kinase inhibitors (TKI) such as Sorafenib, Sunitinib, and Pazopanib; mTOR inhibitors such as Everolimus, Temsirolimus; and anti-VEGF-humanized antibody Bevacizumab failed to deliver long-term survival benefits.^51–53^ A recently introduced programmed death-1 (PD-1) antibody-based immune checkpoint inhibition therapy provided objective response in a subset of patients (~25%); however, the improvement in median overall survival is not drastically improved.^54^ Lately, a combination of two immune checkpoint inhibitors, Nivolumab and Ipilimumab, eclipsed the efficacy of Sunitinib in a phase III trial and has been approved as the new standard of care treatment for intermediate- and poor-risk patients with previously untreated advanced RCC.^55^ Moreover, the combination of Axitinib with either Avelumab (a programmed death ligand 1 inhibitor) or Pembrolizumab (a programmed death-1 inhibitor) exhibited superior progression-free survival and objective response rate over Sunitinib in two distinct large phase III trials.^56,57^ Significant improvement in overall survival was also observed in patients treated with Axitinib and Pembrolizumab. Expectedly, both the combinations have been approved for the first-line treatment in advanced RCC. However, treatment-related adverse events were observed in a majority of patients treated with these combination therapies. Therefore, an unmet clinical need exists for novel treatment strategies as well as targeted delivery systems for advanced metastatic RCC.

The phosphatidyl-inositol-3 kinase (PI3K)/Akt/mTOR pathway is a signaling axis responsible for various crucial functions in cellular homeostasis including protein synthesis, glucose metabolism, survival, migration, and angiogenesis.^58,59^ It has also been implicated in the pathogenesis of various cancers including RCC.^60^ mTOR inhibitors such as Temsirolimus and Everolimus have been approved for first- and second-line treatments for RCC, respectively. We selected Everolimus in our study since it was approved for second- and third-line therapy in patients with advanced RCC. Previously, Everolimus had been shown to inhibit cell growth, migration, and invasion in RCC cell lines in vitro.^61,62^ Everolimus manifested antiangiogenic properties as well, but in a different way than other VEGFR-TKIs.^63^ Everolimus has been shown to inhibit VEGF expression in tumor cells.^61,64^ Interestingly, no significant clinical benefit of Everolimus was found in first-line setting either as a monotherapy or in combination with Bevacizumab.^65^ However, a recent Phase II study demonstrated that a second-line combination therapy with Everolimus and Lenvatinib (a novel TKI) commanded a substantial increase in progression-free survival and overall survival compared to monotherapy with Everolimus.^66^ Hence, it is prudent to postulate that a carefully designed combination strategy with Everolimus as one of the agents may prove to be beneficial.

Recently it has been proposed that the role of VEGF in cancer is not all about angiogenesis and vascular permeability. Several recent articles including ours confirmed the presence of a VEGF-mediated autocrine signaling mechanism in tumor cells that contributes to tumorigenesis and drug resistance by imparting stem-cell like features to cancer cells.^15–19^ Interestingly, all these articles point towards the VEGF/NRP1 axis to be the major player behind this recently discovered angiogenesis-independent function of VEGF. As NRP1 lacks a kinase domain, downstream effector molecules including Ras^16^ and β-catenin^18^ have been proposed to be involved in this intriguing signaling pathway. Nonetheless, the VEGF/NRP signaling axis became a prime target overnight for anticancer therapy because it can impart stemness and drug resistance to cancer cells.

A number of peptide-based competitive inhibitors of VEGF-NRP1 binding were developed that were able to inhibit downstream VEGF signaling pathways.^32–35^ EG00229, the first small-molecule competitive inhibitor of VEGF-NRP1 binding,^36^ has been designed from previously developed bicyclic peptide EG3287 that resembles the C-terminal of VEGF.^34^ EG00229 has been shown to bind to the b1 domain of NRP1 leading to the disruption of VEGF-NRP1 binding.^36^ This, in turn, results in the inhibition of VEGF/VEGFR2/NRP1-mediated proangiogenic signaling in endothelial cells. In addition, disruption of VEGF-NRP1 axis inhibits the tumor cell autocrine signaling via regulating the expression and function of various downstream effector molecules including Ras and β-catenin.^16,18^ Therefore, it is not at all surprising that EG00229 demonstrates combined antiangiogenic and antitumor activity to delay tumor progression in multiple cancer models.^18,36,37^ Moreover, the immune-modulatory function of EG00229 has been suggested in a recent article.^39^

Hence, in the present work, we hypothesized that targeting the VEGF signaling in ccRCC via a bifurcated approach by combining Everolimus and EG00229 in a tumor-targeted liposomal formulation will be beneficial. In addition to its antiproliferative effect, Everolimus will inhibit VEGF synthesis, thereby reducing VEGF levels in tumor microenvironment. At the same time, EG00229 will disrupt VEGF–NRP1 axis leading to the inhibition of VEGF/VEGFR2/NRP1-mediated proangiogenic signaling pathways in endothelial cells and VEGF/NRP1/Ras-mediated autocrine activation of tumor cell growth. Therefore, our strategy enjoys a significant difference from the combination of Everolimus and Bevacizumab that did not improve the patient outcome remarkably. Bevacizumab works by binding to VEGF that is secreted in the tumor microenvironment and inhibiting its angiogenic activity. On the contrary, EG00229 binds to the b1 domain of NRP1 and disrupts the angiogenic signaling in tumor-endothelial cells as well as autocrine signaling in tumor cells as mentioned above. In addition, immune-modulatory effect of EG00229 may act against the immune-suppressive function of Everolimus,^67^ thus promoting antitumor immunity. Consequently, the combination of Everolimus and EG00229 is anticipated to be superior to the combination of Everolimus and Bevacizumab, although this concept needs further evaluation.

We further postulated that the tumor-targeted delivery will require lower doses of Everolimus and EG00229. Indeed, we attained notable tumor inhibition while using lower doses of these drugs than are usually administered. We used 1 mg/kg Everolimus three times a week whereas it is normally administered daily via oral route at 1–5 mg/kg/day.^68^ Similarly the typical dose of EG00229 is 10 mg/kg three times a week via intraperitoneal route^19^ but we have only used 300 µg/kg EG00229 three times a week. Of importance, we only doubled the dose for the highly aggressive Renca model but decreased the frequency of administration. We started the treatments with larger initial tumor volumes than those typically used in the animal studies reported in literature and still achieved significant tumor growth inhibition. We also demonstrated the immune-modulatory effect of EG-L in an immune-competent mouse model of ccRCC. CXCR3-associated chemokine ligands CXCL9, CXCL10, and CXCL11 demonstrate pleotropic roles in immunity and angiogenesis and correlate with poor prognosis in patients with ccRCC.^69,70^ Increased expressions of these chemokines were observed in tumors compared to the normal kidney tissues.^71^ These CXCR3 ligands are also known to recruit regulatory T cells that lead to the suppression of effector T cells which may be one of the reasons of the poor patient outcome.^72–74^ In addition, increased expression of these chemokines has been associated with metastasis in RCC.^75^ Hence, these ligands exert a paracrine effect on tumor microenvironment as well as an autocrine effect in the tumor cells. Similarly TGF-β signaling has been implicated in RCC progression and metastasis.^76,77^ Hence, by reducing the levels of these ligands, EG-L may improve the therapeutic response in patients. Finally, our treatment strategy demonstrated promising reductions in the metastatic burden that may prove to be beneficial for ameliorating the poor survival in patients with advanced metastatic ccRCC.

In summary, we demonstrated that simultaneous tumor-targeted inhibition of mTOR (with Everolimus) and VEGF/NRP1 axis (with EG00229) with the help of a tumor-targeting liposomal formulation was able to induce remarkable inhibition of tumor growth in two different ccRCC xenografts and in a highly aggressive syngeneic mouse model of kidney cancer. In addition, this treatment regimen substantially inhibited the lung metastasis. Taken together, our data establish that a tumor-targeted liposomal formulation encapsulating Everolimus and EG00229 may offer a prospective therapeutic option towards the arsenal of currently available treatment regimens for combating metastatic ccRCC.

## Methods

### Reagents

DOPC and DSPE-(PEG)_2000_-OMe were purchased from Avanti Polar Lipids and Nanosoft Polymers, respectively. Cholesterol was purchased from Sigma. Everolimus and EG00229 were obtained from LC laboratories and Tocris Bioscience, respectively. Ki67 (ab15880), PD-L1 (PA5-88105), β-actin (A2228), and YM1 (60130) antibodies were purchased from Abcam, Invitrogen, Sigma, and STEMCELL Technologies, respectively. The goat-anti-mouse PD-1 antibody was a kind gift from Dr. Keith L. Knutson (Mayo Clinic).

### Cell culture

786-O and A498 cell lines were obtained from American Type Culture Collection (ATCC). Renca cell line was a kind gift from Dr. John A. Copland (Mayo Clinic). No authentication of the cell lines was done by the authors. 786-O and A98 cell lines were maintained in Dulbecco’s modified Eagle's medium (DMEM) and RPMI-1640 medium was used for maintaining Renca cell line. Both the media were supplemented with 10% FBS and 1% penicillin–streptomycin (Invitrogen) and cells were cultured at 37 °C in a humidified atmosphere with 5% CO_2_. Cells from 85% to 90% confluent cultures were used in all of the experiments.

### Synthesis of tumor-targeting-peptide (TTP)-conjugated lipopeptide

Fmoc-strategy-based solid phase peptide synthesis method was used to synthesize the TTP (a tumor-targeting-peptide with a proprietary sequence)-conjugated lipopeptide used in this study.^78^

### Preparation of empty liposomes

A modified ethanol injection method was used to prepare the liposomes.^78^ Required amounts (Table 1) of TTP-conjugated lipopeptide, phospholipids, and cholesterol were dissolved in ethanol and the solution was warmed at 65 °C for 5 min. The solution was then slowly injected into milli-Q water pre-heated to 65 °C while stirring the mixture continuously which led to the spontaneous formation of liposomes. The liposomal solution was stirred at room temperature for an additional 15 min. Finally, rotary evaporation was used to remove ethanol and a part of water under reduced pressure and volume was adjusted with milli-Q water. The liposomes were stored at 4 °C until further use.

### Preparation of drug-loaded liposomes

To prepare the dual-drug-loaded liposomes, EG00229 and Everolimus were included in the aqueous phase and the ethanolic solution of lipids, respectively. The single drug-loaded liposomes were prepared via similar methods where only the drug of choice was used. Following removal of ethanol, Amicon ultra centrifugal filters (3 kDa molecular weight cut-off) were used to remove any unentrapped drugs. The liposome concentrates thus obtained were diluted back to original volumes with milli-Q water and were stored at 4 °C until further use.

### Liposome size and zeta potential analysis

Dynamic light scattering (DLS) measurements were performed using a Malvern Zetasizer (Malvern, UK) to determine the mean hydrodynamic diameter and zeta potential of empty and drug-loaded liposomes after sample dilution with deionized water. Triplicate measurements were performed in samples diluted with milli-Q water at 25 °C.

### Analysis of DLE and EE

EE and DLE were calculated by estimating the amount of entrapped drugs according to previously published procedures. Briefly, during preparation of drug-loaded liposomes, the unentrapped drugs (UE_drug_) were collected in the filtrate obtained from the amicon ultra centrifugal filter with a cut-off size of 3 kDa. Then, absorbance values at *λ*_max_ of respective drugs in the filtrates were measured and compared with respective standard curves to determine UE_drug_ amounts. The encapsulated drug (E_drug_) amount was obtained by deducting UE_drug_ from total drug (T_drug_) amount. Finally, EE was calculated as the percentage of E_drug_ to T_drug_ while DLE was expressed as the percentage of E_drug_ to the total lipid amount (T_lipid_).

### Animals used in the study

Six- to eight-week-old male SCID and Balb/c mice were obtained from in-house breeding and housed in the institutional animal facilities. All animal experiments described in this study were performed under Mayo Clinic Institutional Animal Care and Use Committee (IACUC) approved protocols.

### In vivo biodistribution of liposomes

5 × 10^6^ 786-O or A498 cells resuspended in 100 µL of 50% matrigel in PBS were subcutaneously implanted into the right flank of six- to eight-week-old male SCID mice. After 6–7 weeks, when the average size of tumors reached 300–500 mm^3^, either control (CL) or TTP-conjugated (TL) liposomes loaded with IR-780-Dye were intravenously administered. Fluorescence imaging was performed in live mice under anesthesia using an IVIS imager 24 and 48 h post administration. Finally, mice were euthanized to harvest the tumors and major organs for ex vivo imaging.

### In vitro cytotoxicity assay

Approximately, 5 × 10^3^ 786-O or A498 cells per well were seeded in 96-well plates and allowed to settle for 18–24 h. Then, cells were treated with increasing concentrations of L, E-L, G-L, and EG-L diluted in respective media and incubated for an additional 72 h (*n* = 4 wells per concentration). Cell viability was determined with Celltiter 96 Aqueous One Solution Cell Proliferation Assay kit (Promega) following the manufacturer’s protocol. Briefly, cells were washed once with PBS after aspirating the media containing the treatments from the wells. Then 100 μL fresh media supplemented with 20% One Solution reagent was added to each well and the plate was incubated for 30 min at 37 °C. Finally, absorbance at 492 nm was determined using Spectramax i3x. Percentage viability is calculated using the formula: viability (%) = 100 × (*A*_Treated_ − *A*_Blank_)/(*A*_Untreated_ − *A*_Blank_).

### In vivo tumor regression experiment

The in vivo tumor regression efficacy of the drug-loaded liposomes was analyzed in an SMT using subcutaneous 786-O xenografts (*n* = 1 per treatment group). Empty liposome (L), liposome containing Everolimus (E-L), EG00229 (G-L), and a combination thereof (EG-L) were intravenously administered three times a week into mice bearing ~300–500 mm^3^ tumors. The liposome amounts for each treatment was determined to keep the Everolimus amount administered in E-L and EG-L treated groups constant at 20 µg/mouse/dose. Tumors were measured weekly with calipers and tumor volumes were calculated using the formula: volume = 0.5 × *a* × *b*^2^ where *a* and *b* are the longest and shortest diameter, respectively. Tumor growth curves were obtained by plotting tumor volumes against time. Finally, mice were sacrificed to harvest the tumors along with liver, kidney, and spleen for immunohistochemistry. A similar experiment was performed in A498 xenografts (*n* = 1 per treatment group). In order to validate the results obtained from the SMT, we analyzed the efficacy of EG-L in larger cohorts of 786-O tumor bearing mice (*n* = 5 per treatment group). In addition, we also analyzed the efficacy of EG-L in Renca syngeneic mouse ccRCC model in Balb/c mice (*n* = 5 per treatment group), a highly aggressive tumor that accurately mimics the growth pattern of human ccRCC. Due to the aggressive tumor growth, we started treatment at ~120 mm^3^ starting tumor volume and increased the dose of Everolimus to 40 µg/mouse/dose but reduced the frequency of administration to twice a week in this experiment. In addition, anti-PD-1 antibody and a small-molecule inhibitor of PD-1/PD-L1 interaction were used in two separate SMT experiments (*n* = 1 per treatment group) in the Renca model to analyze any additive or synergistic effect on EG-L treatment.

### Immunohistochemistry

Tumors, livers, kidneys, and spleens were harvested and fixed in neutral-buffered 10% formalin at room temperature for 24 h. Then they were embedded in paraffin and 5-µm-thick sections were cut for preparing slides. Hematoxylin and eosin (H&E) and Ki67 staining (1:1000) were performed in deparaffinaized slides as per the manufacturer’s instructions (DAB 150; Millipore). For Renca tumor sections, YM1 immunostaining (1:100) was also performed. Slides were stained with stable diaminobenzidine and counterstained with hematoxylin. Finally, slides were digitized using an Aperio AT2 slide scanner (Leica) and analyzed using Imagescope software (Leica).

### Quantitative polymerase chain reaction (qPCR)

Total RNA was isolated from a portion of the tumors using RNeasy Plus Mini Kit (Qiagen) as per the manufacturer’s instructions. Reverse transcription was performed using iScript™ cDNA Synthesis Kit (Bio-Rad). Primers were designed using Ensembl genome browser 96 (Supplementary Table S1). Finally, qPCR was performed for the specified targets using Power SYBR Green PCR Master Mix (Applied Bioscience) in an ABI 7500 Real-Time PCR System (Applied Bioscience).

### Immunoblot analysis

Lysates were prepared from homogenized tumor samples using NP-40 lysis buffer supplemented with a protease inhibitor cocktail. Protein concentrations of the lysates were measured by Bradford assay. An equal amount of proteins from each sample was subjected to SDS-PAGE and transferred to polyvinyl difluoride membranes followed by immunoblotting with PD-L1 (1:1000), PD-1 (1:500), and β-actin (1:10,000) antibodies and respective secondary antibodies (1:10,000). Enzyme-linked chemiluminescence was used to detect antibody-reactive bands in Chemidoc MP (Bio-Rad). Quantification of band intensities was performed using Image Lab (Bio-Rad). Blots from same experiments were used for presentation. The uncropped scans of the blots are provided in Supplementary Fig. S5.

### Statistical methods

The double-sided unpaired two-tailed *t*-test was utilized to determine the probability of significant differences between treatment groups where applicable. Statistical significance was defined as *P < 0.05, **P < 0.01, and ***P < 0.001, respectively. Error bars are indicative of calculated SD values.

## Supplementary information

Supplementary Material

nr-reporting-summary

## Data Availability

All data supporting the conclusions of this study are available within this article and the supplementary information.
