# Supplementary material for: Synchronous inhibition of mTOR and VEGF/NRP1 axis impedes tumor growth and metastasis in renal cancer
Source: NPJ Precis Oncol. 2019 Dec 5;3:31. doi: 10.1038/s41698-019-0105-2 (PMC6895165; doi:10.1038/s41698-019-0105-2)
Supplement: Supplementary file 1 — Supplementary Material [file 41698_2019_105_MOESM1_ESM.pdf]

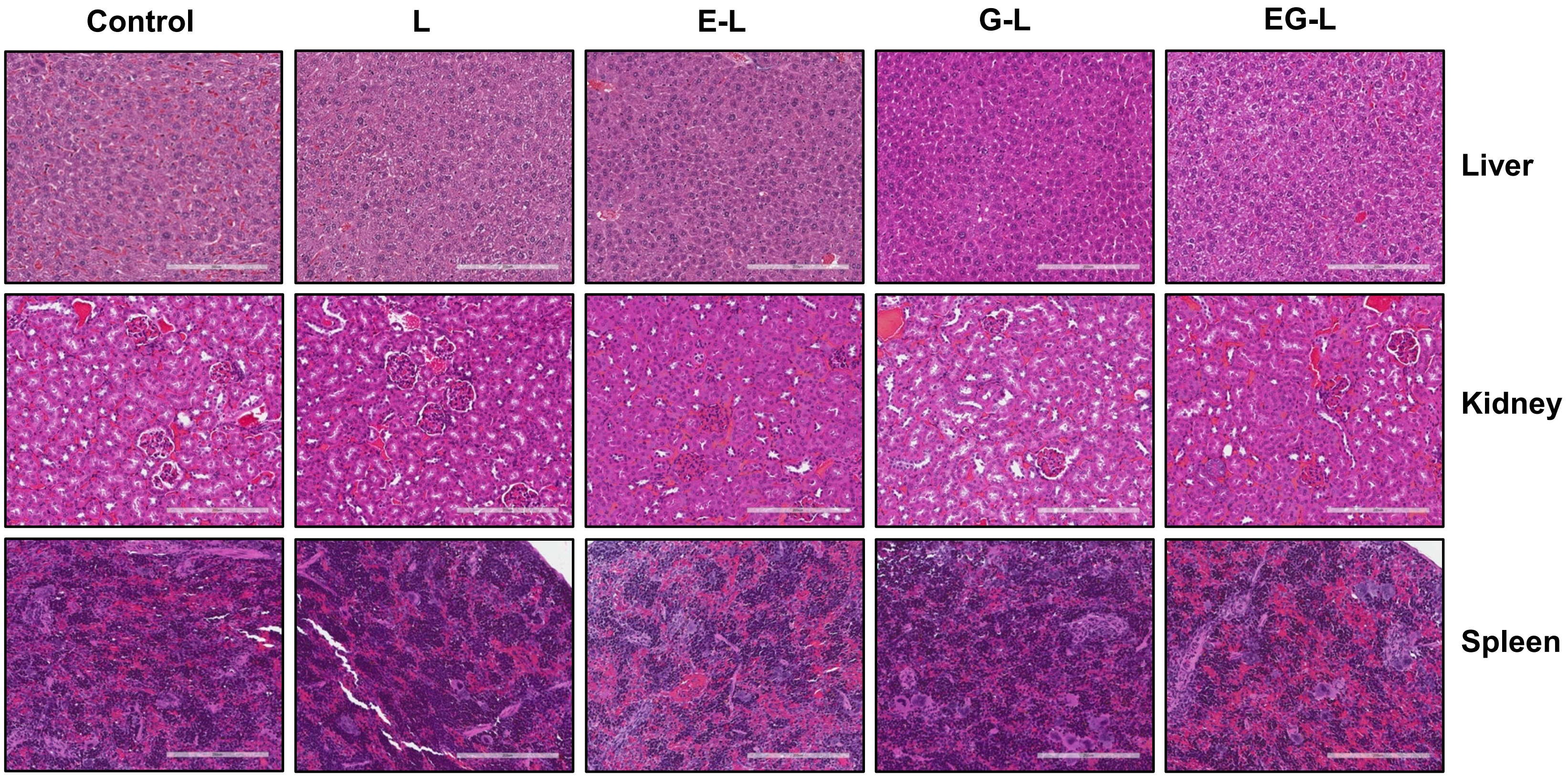

Supplementary Figure S1: H&E staining of liver, kidney and spleen collected from mice bearing 786-O xenografts. No significant morphological changes were observed due to treatment. Bar length = 200 µm.

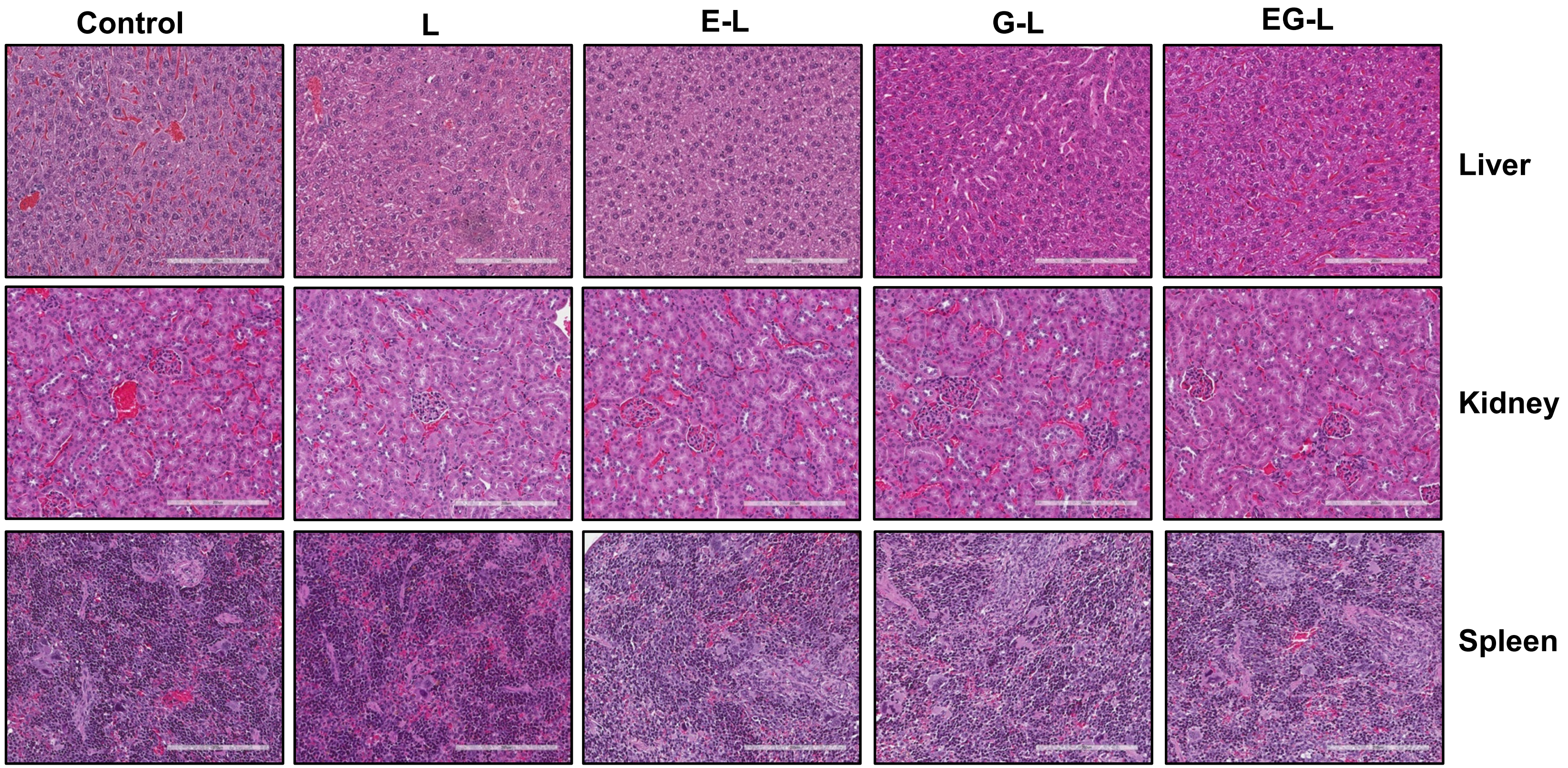

Supplementary Figure S2: H&E staining of liver, kidney and spleen collected from mice bearing A498 xenografts. No significant morphological changes were observed due to treatment. Bar length = 200 μm.

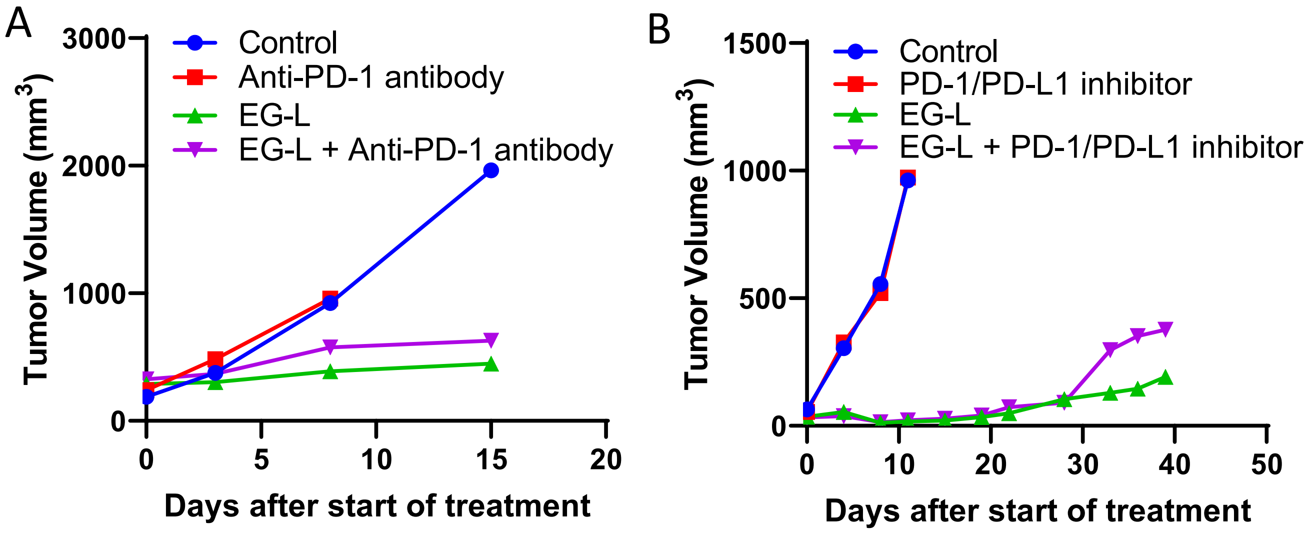

Supplementary Figure S3: Single mouse trial demonstrating the anti-PD-1 therapy resistance of Renca mouse model used in this study. Both anti-PD-1 antibody and PD-1/PD-L1 small molecule inhibitor did not show any tumor growth inhibitory effect. EG-L did not show any additive or synergistic effect when combined with anti-PD-1 therapy either. In fact, anti-PD-1 therapy seemed to exert slight antagonistic effect to EG-L treatment in the long run. (n= 1 mouse per treatment group)

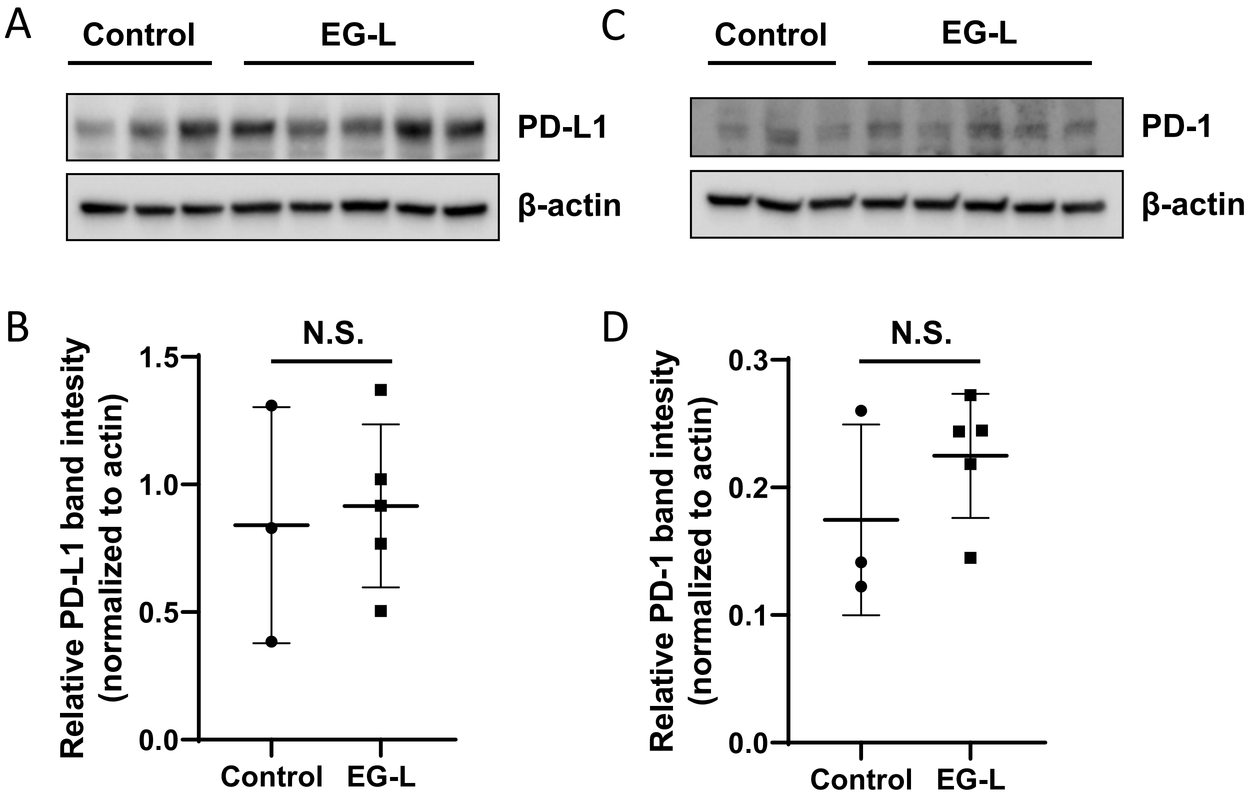

Supplementary Figure S4: Effect of EG-L treatment on the expression of PD-1 in Renca tumors. EG-L treatment did not significantly change PD-L1 (A,B) or PD-1 (C,D) expression in the Renca tumors compared to the vehicle treated tumors. N.S. means not significant. (n = 3 tumors for control and 5 tumors for EG-L)

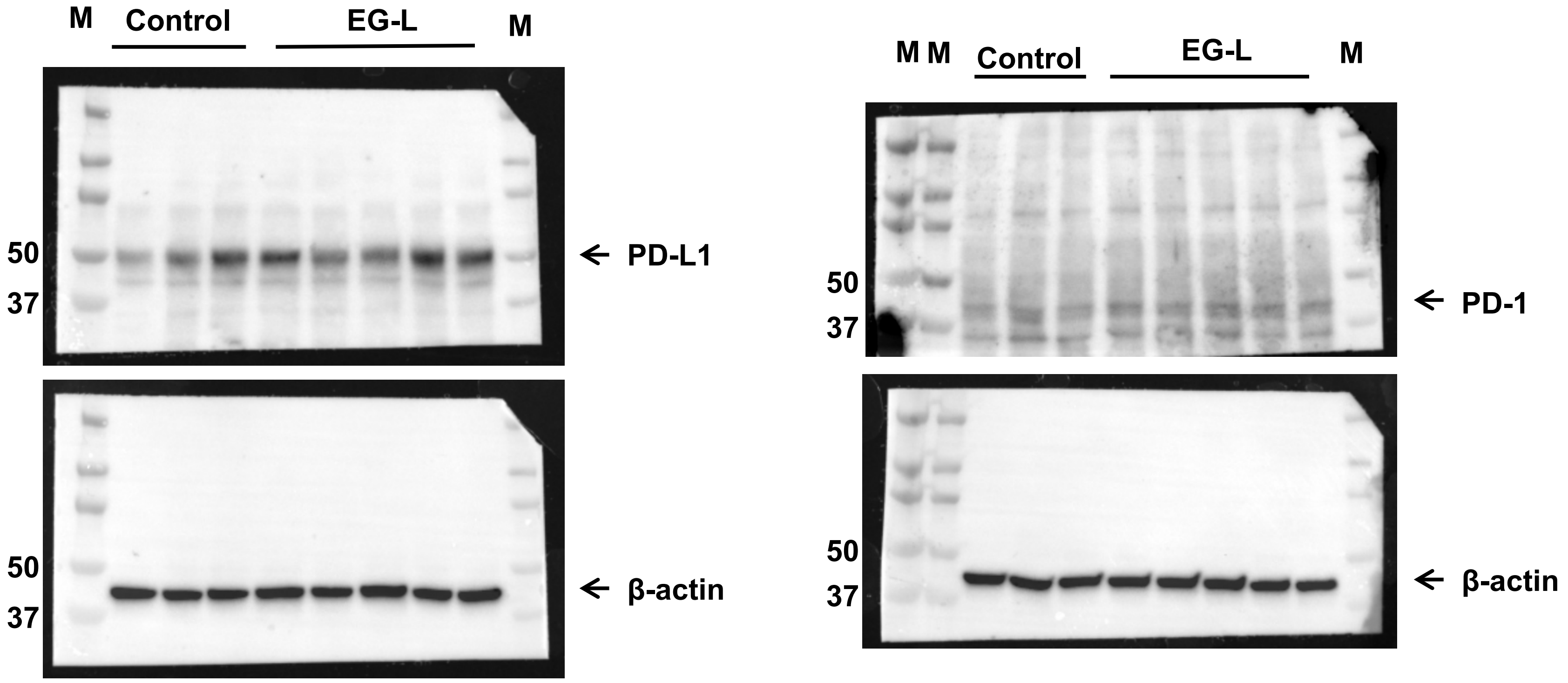

Supplementary Figure S5: Uncropped blots showing the bands of the respective target proteins used in Figure S4. The merged images show the bands along with the markers (M).

Target	Forward Primer	Reverse Primer
CXCL9 (m)	5′-TGTGGCTGTAGCTTAGTGGC-3′	5′-GCCAATGCCTGGTGTGTAAC-3′
CXCL10 (m)	5′-ATGACGGGCCAGTGAGAATG-3′	5′-CTCCCCAGACCACCATTTCC-3′
CXCL11 (m)	5′-AGCCACCCAAAGCTAGGAAC-3′	5′-CCTGCCTCCCTCACATAACC-3′
TGFB1 (m)	5′-TCCTAACCCCAGAGGTGGAC-3′	5′-CCGAAATTCTGCGTCTGCTG-3′
ACTB (m)	5′-GGCACCACACCTTCTACAATG-3′	5′-GGGGTGTTGAAGGTCTCAAAC-3′

Supplementary Table S1: Primer Sequences used in this study.
